# Liver Fat Content and Body Fat Distribution in Youths with Excess Adiposity

**DOI:** 10.3390/jcm7120528

**Published:** 2018-12-07

**Authors:** Robinson Ramírez-Vélez, Mikel Izquierdo, Jorge Enrique Correa-Bautista, María Correa-Rodríguez, Jacqueline Schmidt Rio-Valle, Emilio González-Jiménez, Katherine González-Jiménez

**Affiliations:** 1 Centro de Estudios Para la Medición de la Actividad Física CEMA, Escuela de Medicina y Ciencias de la Salud, Universidad del Rosario, Bogotá 111221, Colombia; jorge.correa@urosario.edu.co (J.E.C.-B.); katherine.gonzalez@docentes.umb.edu.co (K.G.-J.); 2 Department of Health Sciences, Public University of Navarre, Navarrabiomed, IdiSNA, CIBER de Fragilidad y Envejecimiento Saludable (CB16/10/00315), Tudela, 31006 Navarre, Spain; mikel.izquierdo@gmail.com; 3 Departamento de Enfermería, Facultad de Ciencias de la Salud, Avda. De la Ilustración, 60, University of Granada, 18071 Granada, Spain; macoro@ugr.es (M.C.-R.); jschmidt@ugr.es (J.S.R.-V.); emigoji@ugr.es (E.G.-J.); 4 Grupo de Ejercicio Físico y Deportes, Facultad de Salud, Programa de Fisioterapia, Universidad Manuela Beltrán, Bogotá 110231, Colombia

**Keywords:** fatty liver, adiposity, youths, diet, cardiorespiratory fitness

## Abstract

This study had two main objectives: To examine the association between body fat distribution and non-alcoholic fatty liver disease (NAFLD) and liver fat content, and to determine whether the relationship between NAFLD and regional body fat distribution, with respect to liver fat content in youths with excess adiposity, is independent of cardiorespiratory fitness (CRF) and a healthy diet. Liver fat content (controlled attenuation parameter (CAP)), body fat distribution (body mass index (BMI) *z*-score, waist circumference, waist-to-height ratio, fat mass/height, body fat percentage, total fat mass, android-to-gynoid fat mass ratio, visceral adipose tissue (VAT), and lean mass index, determined by dual-energy X-ray absorptiometry (DXA)), CRF (20-m shuttle-run test), and healthy diet (adherence to the Mediterranean diet by KIDMED questionnaire) were measured in 126 adolescents (66% girls) aged between 11 and 17 years. Participants were assigned to two groups according to the presence or absence of hepatic steatosis (CAP values ≥225 dB/m or <225 dB/m of liver fat, respectively). Considering the similar total fat values for the two groups (>30% by DXA), youths with NAFLD had higher fat distribution parameters than those without NAFLD, regardless of sex, age, puberty stage, lean mass index, CRF, and healthy diet (*p* < 0.01). In the non-NAFLD group, the association between hepatic fat and fat distribution parameters presented a similar pattern, although the association was statistically insignificant after adjusting for a potential confounding variable (*ps* > 0.05), except for the case of VAT. Body fat distribution parameters were higher in youths with NAFLD compared to those without NAFLD. Additionally, body fat distribution showed a significant association with liver fat content as assessed by CAP in youths with NAFLD independent of CRF and adherence to the Mediterranean diet, supporting the notion that upper body fat distribution might play a pivotal role in the development of NAFLD in adolescents. These results may have implications for the clinical management of youths with excess adiposity given the high prevalence of NAFLD in children and young adults.

## 1. Introduction

Non-alcoholic fatty liver disease (NAFLD), a well-recognized cause of chronic liver disease, is a common clinical condition characterized by abnormal triglyceride accumulation in the liver [1]. Estimates of the incidence and global prevalence of NAFLD in western and developing countries are worrying when considering the parallel burden of obesity and its metabolic complications [2,3]. A recent meta-analysis showed that obese individuals had a 3.5-fold increased risk of developing NAFLD, supporting a relationship between body mass index (BMI) and NAFLD risk [2]. Furthermore, it has been reported that up to one-third of overweight adolescents present with NAFLD [4]. The rate of NAFLD varies between ethnic and racial groups, and in Latin America the prevalence ranges from 7.6% to 34.2% [5].

The pathogenesis and progression of pediatric NAFLD remains unclear, but an unhealthy lifestyle, including sedentarism and a poor diet, could be responsible for the high prevalence of NAFLD since it is known to increase with obesity, metabolic syndrome, and type 2 diabetes [6,7]. Thus, there is growing evidence to support that pediatric NAFLD is closely related to excess adiposity and its metabolic consequences [8]. Of particular note are visceral adipose tissue (VAT) and subcutaneous adipose tissue (SAT) levels, which are believed to play a significant role in increased liver fat in youths [9].

Adipose tissue has traditionally been considered to be a simple triacylglycerol storage organ. However, over the last decade several publications have stimulated research into its endocrine functions as the synthesis and secretion of several hormones [10]. Adipose tissue is involved in a range of processes, including control over nutrient uptake, insulin sensitivity, and inflammatory mediators [10]. Excess adiposity, especially abdominal obesity, is associated with peripheral insulin resistance, which in turn leads to metabolic syndrome, metabolic inflexibility, dyslipidemia, hyperglycemia, hypertension, and other metabolic abnormalities [11]. Recent data have shown that body fat distribution (i.e., fat mass and android-to-gynoid fat ratio) could modulate the action and metabolism of the liver and skeletal muscle, leading to an increased risk of cardiovascular disease [12]. 

In the same line, regional body fat distribution, as measured by VAT and SAT area, has been proposed as a determinant of NAFLD irrespective of general obesity [9,13]. Whereas VAT involves an active endocrine organ that regulates metabolism and inflammation, SAT may act as a “metabolic sink” and protect against the development of metabolic abnormalities [14]. Given that overweight or obese adolescents have a greater NAFLD risk profile (i.e., hyperinsulinemia and lower insulin sensitivity) than their normal-weight peers [15], it would be useful to learn whether the association between body fat distribution parameters and NAFLD is independent of lifestyle factors closely associated with obesity such as cardiorespiratory fitness (CRF) or a healthy diet. In addition, to date, most previous studies have used simple anthropometric parameters such as weight, height, BMI, or skinfold thickness to investigate the association between body fat distribution and NAFLD, whereas only a few studies have used dual-energy X-ray absorptiometry (DXA) to examine body composition measurements [16,17].

In this context, the aims of the present study were three-fold: (i) To examine body fat distribution parameters between youths with excess adiposity in the presence and absence of NAFLD; (ii) to study the association between body fat distribution parameters and liver fat content by determining the controlled attenuation parameter (CAP), which is an indicator of hepatic fat deposition; and (iii) to investigate whether the relationship between body fat distribution parameters and liver fat content is independent of lifestyle factors related to adipose tissue such as CRF or a healthy diet (optimal adherence to the Mediterranean diet).

## 2. Experimental Section

### 2.1. Study Design, Setting, and Participants

The present cross-sectional study was developed under baseline analysis of the clinical trial Exercise Training and Hepatic Metabolism in Overweight/Obese Adolescents (HEPAFIT), ClinicalTrials.gov Identifier: NCT02753231. Details of background and design methods of the HEPAFIT Study have been previously published elsewhere [18]. For the current purposes, baseline data from 126 adolescents (66% girls) aged between 11 and 17 from Bogotá, Colombia, partook in this study. The following inclusion criteria were adopted: Primary overweight or obese status, based on the age- and sex-specific BMI cutoff values of the International Obesity Task Force (IOTF) guidelines [19], or excess of adiposity (body fat >30% by dual-energy X-ray absorptiometry (DXA)), and inactivity (no participation in exercise more than once per week in the previous six months). All participants were informed of the study’s goals, and written informed consent was obtained from participants and their parents or legal guardians. The protocol of the study was reviewed and approved by the Medical Research Ethics Committee of the University of Rosario (ID CEI-ABN026-000140) and conducted in accordance with the Declaration of Helsinki.

### 2.2. Physical Examination

Weight (kg) was measured with an electronic scale (Model Tanita^®^ BC-418^®^, Tokyo, Japan), and height (cm) was measured with a stadiometer (Seca^®^ 206, Hamburg, Germany), measured in duplicate following standard protocols. Body mass index (BMI) *z*-score was calculated using WHO Anthro-Plus program (AnthroPlus software^®^, version 1.0.4, World Health Organization, Geneva, Switzerland, 2011). Waist circumference was obtained in the standing position, at the middle point between the anterior iliac crest and lower border of the rib, using a tape measure. Pubertal stage was recorded by self-report according to Tanner and Whitehouse [20]. Waist-to-height ratio (WtHR) was calculated as the ratio of waist circumference to height (both in cm). Anthropometric variables were measured by a Level 2 expert certified by the International Society for the Advancement of Kinanthropometry. The same trained investigator made all anthropometrics measurements.

### 2.3. Body Composition

Fat mass/height (kg/m^2^), body fat (%), total fat mass (kg), android/gynoid fat mass (kg), VAT (cm^3^), and lean mass index (calculated by dividing lean mass by the square of the height), were measured by DXA using the Hologic Horizon DXA System^®^ (Quirugil, Florida, MI, USA) with Discovery software, version 12.3 (Bellingham, WA, USA). The DXA equipment was calibrated at the start of each testing day by using a lumbar spine phantom as recommended by the manufacturer and was completed following the same protocol by the same researcher within each study. All subjects were assessed for all included measures related to physical examination and body composition in the same day.

### 2.4. Liver Fat Content

The FibroScan^®^ 502 Touch device (Echosens, Paris, France), with the M probe placed on the skin between the ribs over the right lobe of the liver, was used to capture the CAP, as a surrogate marker of the deposit of fat in the liver. The detailed protocol of the measurement and calculations has been published elsewhere [21]. Thereafter, participants were categorized according to Desai et al. [22] into two groups according to the presence or absence of hepatic steatosis (CAP values ≥225 dB/m or <225 dB/m of liver fat, respectively). CAP has been proposed as a highly sensitive, noninvasive, and accurate technique for assessing liver fat accumulation [23]. CAP has a number of advantages when compared to other common measures: Providing an instantaneous assessment of liver steatosis, quantificational accuracy and ease of performance, as well as inexpensive cost and reproducibility [24,25]. CAP is also an operator-friendly technique with a high inter-rater reliability concordance correlation coefficient value of 0.82 [26]. Comparing CAP to the conventional ultrasound-based Hamaguchi steatosis scoring system, Carvalhana et al. [27] reported a correlation of *r* = 0.75, *p* < 0.001, between ultrasound and CAP. In the same line, Park et al. [28] reported that magnetic resonance proton density fat fraction had a diagnostic area under the receiver operating characteristic curve (AUROC) values of 0.90 for diagnosing stage 2 and stage 3 versus stage 0 and stage 1, and a 0.92 for diagnosing stage 3 versus stage 0–stage 2, whereas CAP AUROC values were 0.70 and 0.73, respectively. Additionally, in a recent meta-analysis of 19 CAP studies with histologic features as reference [29], CAP had overall AUROC values of 0.823 to detect stage 0 versus stage 1–stage 3, 0.865 to detect stage 0 and stage 1 versus stage 2 and stage 3, and 0.882 to detect stage 0–stage 2 versus stage 3 steatosis.

### 2.5. Healthy Diet

Habitual food consumption was assessed using the brief-type self-administered adherence to the Mediterranean diet. The KIDMED index (Mediterranean Diet Quality Index for children and adolescents) was used [30]. The KIDMED index is a scientifically validated survey that is based on 16 questions, is self-administered, and sustains the principles of the Mediterranean dietary patterns, as well as those that undermine it. The final results of the index vary between 0 and 12 points. In this study, we divided participants into two groups, more than or equal to 8 points (ideal healthy diet) and less than or equal to 7 points (non-ideal healthy diet). 

### 2.6. Maximum Oxygen Consumption

CRF (VO_2_peak, mL/kg/min) was assessed by the 20-m shuttle-run test, which has been shown to be valid, reliable, feasible, and safe for the assessment of physical fitness in children and adolescents [31]. We estimated the VO_2_peak according to the number of laps performed, per Léger et al. [32]. 

### 2.7. Statistical Analysis

All statistical analyses were performed using SPSS version 24.0 for Windows (IBM, Armonk, NY, USA), and the significance level was set at *p* < 0.05. The Kolmogorov–Smirnov normality test was conducted before association analysis. Data were expressed as mean ± standard deviation or median (interquartile range) for continuous variables and as frequencies for categorical variables. Due to their skewed distribution, the following variables were log-transformed before analyses: age, waist circumference, waist-to-height ratio, CAP, and VO_2_peak. To aid interpretation, data were back-transformed from the log scale for presentation in the results. Unadjusted differences in descriptive characteristics between youth with or without NAFLD were compared using an independent *t*-test, the Mann–Whitney U-test, or the chi-square test for the continuous and categorical measures. Differences in fat distribution parameters such as weight, BMI *z*-score, waist circumference, WtHR, fat mass/height, body fat percentage, total fat mass, android/gynoid fat mass, and VAT, between youth with or without NAFLD, were examined by analysis of covariance (ANCOVA), adjusting by sex, age, puberty stage, and lean mass index (Model 1); CRF (Model 1 + CRF), and adherence to the Mediterranean diet (Model 3: Model 2 + Mediterranean patterns). 

The associations of liver fat content with fat distribution parameters were examined using regression analyses controlling for sex, age, puberty stage, and lean mass index (Model 1); and additionally for CRF (Model 2) and adherence to the Mediterranean diet (Model 3). In all models, there were no significant interactions by sex (*ps* > 0.1): Hence the ANCOVA and regression analyses were performed for boys and girls together.

## 3. Results

The descriptive characteristics of the participants in the study are shown in Table 1. We did not observe any significant differences in age, puberty stage, VO_2_ max, and Mediterranean diet optimal adherence between youths with or without NAFLD. Participants with NAFLD had higher values of all fat distribution parameters (weight, BMI *z*-score, waist circumference, WtHR, fat mass-to-height ratio, body fat percentage, total fat mass, android-to-gynoid fat mass ratio, and VAT, *ps* < 0.01).

**Figure 1:**
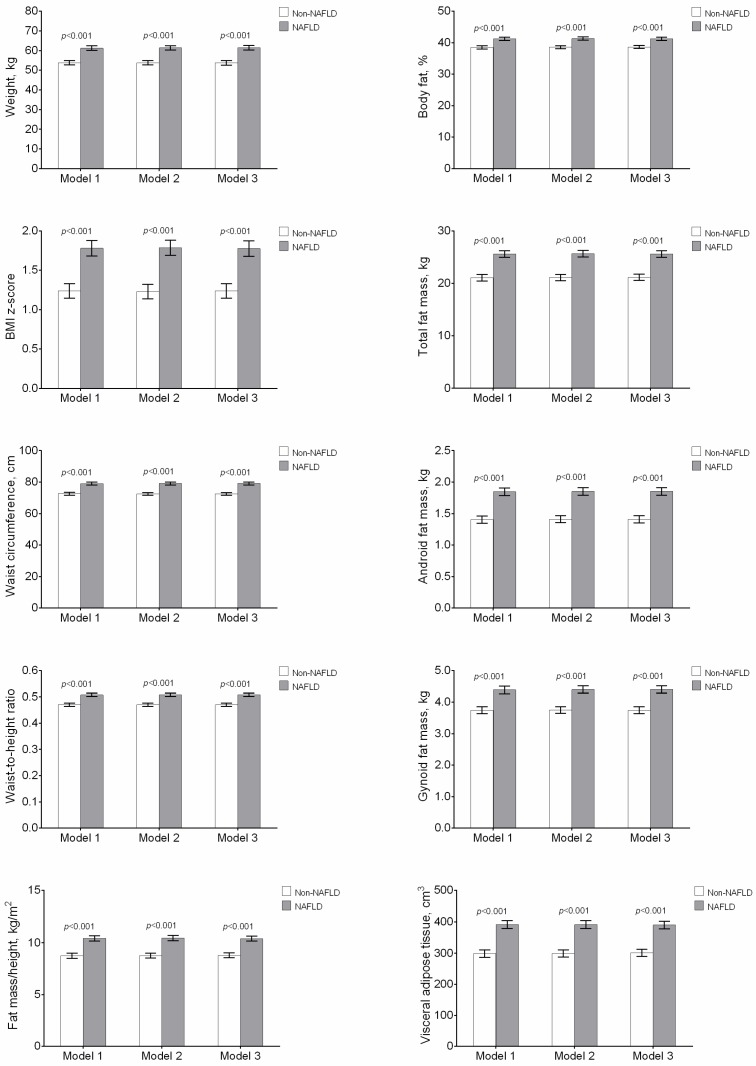
Fat distribution parameters in youth according to the presence or absence of non-alcoholic fatty liver disease (NAFLD). Analysis of covariance (ANCOVA) Model 1: Adjusted for sex, age, puberty stage, and lean mass index; ANCOVA Model 2: Model 1 additionally adjusted for cardiorespiratory fitness (CRF); Model 3: ANCOVA Model 2 additionally adjusted for Mediterranean diet optimal adherence.

The covariance analysis showed that differences in fat distribution parameters between youths with and without NAFLD were independent after adjusting for sex, age, puberty stage, and lean mass index (*p* < 0.001, Model 1, Figure 1), CRF (*p* < 0.001, Model 2, Figure 1), and adherence to the Mediterranean diet (*p* < 0.001, Model 3, Figure 1).

The linear regression showed that higher liver fat content as measured by CAP (NAFLD group) correlated significantly with higher fat distribution parameters independent of sex, age, puberty stage, and lean mass index, except for weight and body fat percentage variables (Model 1, *p* < 0.05, Table 2). The relationships of fat distribution parameters still persisted after adjusting for CRF, except in the case of the weight, body fat percentage, and gynoid fat mass variables (Model 2, *p* < 0.05, Table 2), and for CRF + adherence to the Mediterranean diet (Model 3, *p* < 0.05, Table 2), except in the case of the body fat percentage and gynoid fat mass variables. Lastly, the association of liver fat content as measured by CAP in the non-NAFLD group with fat distribution parameters presented a similar pattern, although the association was statistically insignificant after adjusting for a potential confounding variable (*ps* > 0.05, Models 1 and 3, Table 2), except for the case of the VAT parameter.

## 4. Discussion

We studied the associations between body fat distribution determined by DXA and liver fat content measured by CAP in youths with excess adiposity with or without NAFLD. Our findings demonstrate that youths with excess adiposity were much more likely than those without excess adiposity to have NAFLD assessed by transient elastography, supporting that adiposity is a determinant of NAFLD. Additionally, this study showed for the first time that the direct relationship between body fat variables and liver fat accumulation was independent of CRF and a healthy diet in Latin American youth populations. 

An upper body fat distribution, commonly associated with increased visceral fat, has been related to an abnormal metabolic profile [33]. Whereas some publications have reported that obesity is closely related to NAFLD [34], our study, however, supports two current beliefs: (i) That the correlation between hepatic fat and body fat distribution can be found in adolescence and (ii) that the association between fat distribution and hepatic fat content is independent of lifestyle factors such as CRF or a healthy diet [16].

Our data reveal that there were differences in several body fat parameters including weight, BMI *z*-score, waist circumference, WtHR, fat mass-to-height ratio, percentage of body fat, total fat mass, android fat mass, gynoid fat mass, and VAT between schoolchildren with and without NAFLD after adjusting for several confounding factors. These findings imply that higher body fat distribution might be independently predictive of increased fatty liver risk in adolescents. Similarly, abdominal fat was reported to influence the development of NAFLD in a Korean cohort of obese children [17], whereas Kelishadi et al. [15] showed a correlation between waist circumference and fatty liver biochemical indexes such as alanine aminotransferase (ALT), aspartate aminotransferase (AST), and alkaline phosphatase (ALP) in Iranian children and adolescents. However, it should be noted that Sorrentino et al. [35] suggested that liver enzymes levels cannot be used as surrogate markers of NAFLD. 

We also demonstrated that BMI *z*-score, waist circumference, WtHR, fat mass-to-height ratio, total fat mass, android, and VAT were significantly associated with liver fat content as measured by CAP in youths with NAFLD independently of CRF and adherence to the Mediterranean diet. These findings agree with a recent study that concluded that obese children and adolescents with higher intra-abdominal adipose tissue were more likely to develop NAFLD with independence of possible confounding variables [16]. These results support the “portal free fatty acid” hypothesis that suggests that an expanded visceral fat depot might, through its active lipolysis, expose the liver to high concentrations of free fatty acids, leading to impaired metabolism [36]. Considering that a recent longitudinal study revealed that children with increased adiposity are more likely to develop NAFLD in adulthood [8], our findings are clinically relevant since they sustain that DXA body composition measurements could be significant when assessing the risk of NAFLD in youths with excess adiposity. However, further research is still needed to assess the predictive ability of each body fat parameter in terms of detecting NAFLD.

Previous reports that found an association between being overweight or obese and a higher prevalence of NAFLD either did not account for physical activity patterns or used self-reported measures of physical activity, which led to substantial misclassification [37]. CRF is an objective laboratory measurement that reduces the misclassification bias that often results from self-reported measures of physical activity [38]. In particular, CRF has positive effects on endocrine metabolism and body composition in children and adolescents [39]. Furthermore, high CRF levels are believed to improve insulin sensitivity and increase glucose uptake and therefore could be associated with lower fat levels [37]. Contrastingly, low CRF levels are associated with a higher risk of cardiovascular disease and mortality [40], whereas their influence on fatty liver disease is contentious [41]. In the present study, we did not observe any significant differences between CRF levels for adolescents with or without NAFLD. A lack of physical activity levels was also an important limitation of this study.

In young populations, diet has been proposed to influence liver fat content, although the corresponding data are inconsistent [42]. Here, we did not collate enough dietary data from our participants in order to assess the influence of diet, nor did we observe significant associations between adherence to the Mediterranean diet and liver fat as measured by transient elastography. In any case, we did not detect a substantial difference in adherence to the Mediterranean diet between adolescents with and without NAFLD after taking into account food consumption variables (data not shown).

The pathophysiological mechanisms underlying the interplay between NAFLD and fat distribution remain unknown. Low-grade inflammation, peripheral insulin resistance, and elevated serum levels of free fatty acids, especially fatty infiltration of the liver, may modulate the action and liver metabolism and have been proposed as key contributors to high fat levels in individuals with NAFLD [43]. Furthermore, a previous meta-analysis concluded that subjects with central obesity measured by WtHR had a higher risk of NAFLD than individuals with general obesity, supporting the idea that body fat distribution plays a primary role in the development of a fatty liver [44]. Considering that the liver is a key organ central to carbohydrate and lipid metabolism [45], in cases of upper body fat distribution we could speculate that portal free fatty acid (FFA) concentrations resulting from lipolysis might be significantly greater than arterial FFA concentrations. Therefore, the liver might be exposed to greater amounts of FFA, leading to an increased risk of NAFLD [46]. Additionally, visceral fat might release sufficient IL-6 to increase portal vein IL-6 concentrations, thus affecting hepatic metabolism [47].

This study had several strengths. First, to the best of our knowledge, this is the first study to investigate whether the association between body fat distribution and NAFLD is independent of CRF and a healthy diet in youths, as previous studies have been conducted in adult cohorts. Second, in addition to conventional anthropometric indices such as BMI *z*-score, waist circumference, and WtHR, we also evaluated body fat distribution using a DXA device, which is considered the gold standard measurement for regional body composition due to its excellent accuracy and reproducibility [48]. It should also be noted that highly standardized procedures were developed as part of the HEPAFIT study in order to reduce measurement bias. 

Nevertheless, we must acknowledge that the study had some limitations. As it is a cross-sectional study, we could not infer causality, and further longitudinal studies are needed to investigate the contribution of body fat distribution in the pathogenesis of NAFLD in young subjects. We used liver transient elastography, a highly sensitive, noninvasive, accurate technique for estimating liver fat accumulation [49]. Additionally, although CAP is established as a useful diagnostic method in detecting liver fat content, a relative disadvantage of this technique is the fact that CAP might be affected by body mass index, larger skin capsular distance, age, width of intercostal space, or visceral fat [25]. However, the convenience and noninvasiveness of CAP for pediatric population screening has not lost its appeal, but rather is becoming increasingly necessary. Furthermore, the internal validity of this study could be significantly limited by genetic basis, which was not taken into consideration and could therefore introduce unobserved heterogeneity. However, we did adjust for several potential confounding factors such as sex, age, puberty stage, lean mass index, CRF, and adherence to the Mediterranean diet. Finally, although our sample was larger than previous studies, it was still relatively small, so these findings must be interpreted with caution, and further studies in larger populations are required to validate our preliminary results. 

## 5. Conclusions

In summary, body fat distribution parameters were higher in youths with NAFLD compared to those without NAFLD. Additionally, body fat distribution was significantly associated with liver fat content measured by CAP in youths with NAFLD irrespective of CRF and adherence to the Mediterranean diet, supporting the theory that upper body fat distribution might play a pivotal role in the development of NAFLD in adolescents. These results may have important implications in the clinical management of youths with excess adiposity given the high prevalence of NAFLD in children and young adults. Future longitudinal studies are required to further clarify the role of body fat distribution in the development of NAFLD.

## Figures and Tables

**Table 1:** Descriptive characteristics of participants in the study.

Characteristics	Whole Sample (*n* = 126)	NAFLD (*n* = 67)	No NAFLD (*n* = 59)	*p*-Value
Chronological age, years ^a^	13 (12–15)	14 (12–15)	13 (12–15)	0.984
Puberty stage (I to V), % *	0/15/26/42/18	0/20/25/39/15	0/11/26/44/20	0.165
Weight, kg	57.4 (10.5)	61.3 (11.5)	54.0 (8.0)	<0.001
BMI *z*-score	1.5 (0.8)	1.8 (0.8)	1.2 (0.8)	<0.001
Waist circumference, cm ^a^	74.0 (70.2–79.7)	77.9 (72.9–85.6)	72.1 (69.2–77.3)	<0.001
Waist-to-height ratio ^a^	0.47 (0.44–0.52)	0.49 (0.46–0.55)	0.50 (0.44–0.49)	<0.001
Fat mass/height, kg/m^2^	9.5 (2.0)	10.3 (2.3)	8.8 (1.6)	<0.001
Body fat, %	39.7 (4.4)	40.8 (4.9)	38.7 (3.7)	0.009
Total fat mass, kg	57.7 (10.0)	61.0 (11.0)	54.8 (8.0)	0.009
Android fat mass, kg	3.8 (0.9)	4.2 (1.0)	3.5 (0.7)	<0.001
Gynoid fat mass, kg	9.2 (1.9)	9.7 (2.0)	8.8 (1.7)	<0.001
Visceral adipose tissue, cm^3^	342.6 (104.4)	396.3 (110.6)	294.9 (70.5)	<0.001
Lean mass index, kg/m^2^	13.6 (1.4)	13.2 (1.3)	14.0 (1.5)	0.008
Controlled attenuation parameter, dB/m ^a^	218.5 (197.0–247.5)	249.0 (237.2–277.1)	198 (181.0–210.1)	<0.001
VO_2_peak, mL/kg/min ^a^	37.9 (36.0–39.9)	37.9 (36.1–40.4)	37.9 (36.0–39.6)	0.290
Mediterranean diet optimal adherence, %	31.0	23.7	37.3	0.093

^a^ Median and interquartile range. Significant between NAFLD groups differences (*t*-tests or * chi-squared test *X*^2^). Here, *z*-BMI: *z*-score of body mass index; VO_2_peak: maximal oxygen uptake; PHV: peak height velocity; NAFLD: non-alcoholic fatty liver disease. Equations to estimate VO_2_peak in boys and girls = 31.025 + 3.238 × (*S* × (3.248 × (*A* + 0.1536 × (*S* × *A*)))), where *A* is age and *S* is final speed (*S* = 8 + 0.5 × last stage completed).

**Table 2:** Association of liver fat content by controlled attenuation parameter (CAP) on fat distribution parameters in youth with an excess of adiposity according to the presence or absence of NAFLD groups.

No NAFLD	Liver Fat Content (CAP, dB/m)					
	Model 1		Model 2		Model 3	
	β	*p*-Value	Β	*p*-Value	Β	*p*-Value
Weight, kg	0.167	0.276	0.176	0.248	0.181	0.253
BMI *z*-score	0.088	0.506	0.094	0.469	0.094	0.482
Waist circumference, cm	0.236	0.180	0.221	0.207	0.220	0.212
Waist-to-height ratio	0.215	0.097	0.188	0.151	0.196	0.142
Fat mass/height, kg/m^2^	0.062	0.655	0.056	0.687	0.054	0.703
Body fat, %	0.032	0.813	0.049	0.716	0.050	0.713
Total fat mass, kg	0.104	0.494	0.109	0.476	0.113	0.466
Android fat mass, kg	0.176	0.220	0.175	0.222	0.177	0.222
Gynoid fat mass, kg	0.012	0.938	0.162	0.271	0.027	0.869
Visceral adipose tissue, cm^3^	0.294	***0.041***	0.290	***0.043***	0.293	***0.045***
**NAFLD**						
Weight, kg	0.102	0.564	0.062	0.733	0.368	***0.013***
BMI *z*-score	0.353	***0.009***	0.298	***0.039***	0.295	***0.043***
Waist circumference, cm	0.797	***<0.001***	0.765	***<0.001***	0.763	***<0.001***
Waist-to-height ratio	0.459	***<0.001***	0.423	***0.002***	0.422	***0.003***
Body fat, %	0.218	0.161	0.138	0.413	0.136	0.426
Fat mass/height, kg/m^2^	0.348	***0.016***	0.297	***0.044***	0.296	***0.049***
Total fat mass, kg	0.349	***0.011***	0.307	***0.039***	0.306	***0.042***
Android fat mass, kg	0.377	***0.006***	0.338	***0.018***	0.338	***0.019***
Gynoid fat mass, kg	0.311	***0.037***	0.256	0.107	0.255	0.114
Visceral adipose tissue, cm^3^	0.568	***<0.001***	0.543	***0.001***	0.544	***0.001***

*β* is a standardized regression coefficient. Model 1: Adjusted for sex, age, puberty stage, and lean mass index; Model 2: Model 1 additionally adjusted for CRF; Model 3: Model 2 additionally adjusted for Mediterranean diet optimal adherence. The bold indicates *p*-values <0.05.
